# Net Pain Relief After Palliative Radiation Therapy for Painful Bone Metastases: A Useful Measure to Reflect Response Duration? A Further Analysis of the Dutch Bone Metastasis Study

**DOI:** 10.1016/j.ijrobp.2019.07.009

**Published:** 2019-11-01

**Authors:** Katie Spencer, Galina Velikova, Ann Henry, Paulien Westhoff, Pe ter Hall, Yvette M. van der Linden

**Affiliations:** ∗ Leeds Institute of Medical Research, University of Leeds, Leeds, United Kingdom; † Leeds Cancer Centre, Leeds NHS Teaching Hospitals Trust, Leeds, Leeds, United Kingdom; ‡ Department of Radiation Oncology, Radboud University Medical Center, Nijmegen, The Netherlands; § Edinburgh Cancer Research Centre, University of Edinburgh, Edinburgh, United Kingdom; ‖ Department of Radiotherapy, Leiden University Medical Centre, Leiden, The Netherlands

## Abstract

**Purpose:**

Pain response rates are equivalent after single 8 Gy and fractionated palliative radiation therapy for bone metastases. Reirradiation remains more frequent after a single fraction, although this does not simply reflect pain recurrence. Given the possible role of stereotactic radiation therapy in providing durable pain control, measures of durability are required. Net pain relief (NPR), the proportion of remaining life spent with pain response, may provide this. This study assesses the use of NPR as an outcome measure after palliative radiation therapy for bone metastases.

**Methods and Materials:**

This is a secondary analysis of data collected in the Dutch Bone Metastasis Study, a randomized trial comparing palliative radiation therapy delivered as 8 Gy in a single fraction and 24 Gy in 6 fractions. NPR was assessed by survival cohorts, treatment regimen, and primary diagnoses. The consequences of missing data upon the use of NPR in future studies were considered within sensitivity analyses.

**Results:**

Patients whose pain improved after palliative radiation therapy experienced improvement for 56.6% of their remaining lives. Missing responses in questionnaires mean the range of uncertainty in NPR is 36.1% to 62.1%. When response beyond reirradiation was excluded, NPR after treatments of single-fraction 8 Gy and 24 Gy in 6 fractions was 49.0% and 56.5%, respectively (*P* = .004). Differential willingness to reirradiate may be influencing this outcome. When response beyond reirradiation was included, this difference was not seen (NPR of 55.4% vs 57.7%, respectively [*P* = .191]).

**Conclusions:**

Patients who responded to conventional radiation therapy experienced improved pain control for approximately half of their remaining life. NPR may provide valuable information in assessing pain response durability. Missing data are, however, inevitable in this population. This must be minimized and the consequences recognized and reported. Additionally, reirradiation protocols and the frequency and duration of trial follow-up may have a significant impact upon this outcome, requiring careful consideration during trial design if NPR is to be used in future studies.

**Summary**

Patients who respond to palliative radiation therapy for bone metastases can expect improved pain for approximately 50% of their remaining life. Net pain relief, although rarely reported, can provide valuable information about response durability. This is particularly important when assessing stereotactic radiation therapy outcomes. Limited trial follow-up, long intervals between questionnaires, and missing data may result in significant uncertainty. This latter must be minimized and clearly reported, and its consequences must be assessed where net pain relief is considered.

## Introduction

Palliative radiation therapy for painful bone metastases, alongside analgesia, is the standard of care. Multiple studies have compared different dose fractionation schedules. Response is assessed as the proportion of individuals gaining an improvement of at least 2 points on a 0 to 10 point numerical rating scale without concurrent increase in opioids.^1^ The extent of response is graded as complete pain response (CR; pain score 0, no analgesia increase), partial response (PR; reduction of >1 point in pain score, no analgesia increase), pain progression (PP; increase of >1 point or analgesia increase without improvement of pain score), or no response (NR). In meta-analyses of randomized studies, single-fraction treatment was as effective for pain control as multifraction courses with response rates of approximately 60%.^2^, ^3^, ^4^

In attempting to quantify response durability, studies routinely report reirradiation rates in the absence of difficult-to-collect longitudinal response data. Twenty percent reirradiation rates are seen after single-fraction treatment compared with 8% after multiple-fraction regimens.^2^, ^5^ Reirradiation reflects a composite endpoint, encompassing pain response, but also perceived treatment efficacy and willingness to reirradiate.^5^ On this basis, reirradiation does not provide a simple representation of response durability. In addition, variations in data collection frequency and pain response assessment make comparisons between studies challenging. As such, although no clear dose-response relationship has been demonstrated using conventional radiation therapy, the suggestion has remained that a higher biologically effective dose, potentially delivered using stereotactic radiation therapy, might provide superior levels of pain relief with greater durability.^6^, ^7^, ^8^

International consensus endpoints have increased homogeneity in bone metastasis trial conduct and reporting. These recognize the potential of net pain relief (NPR) as a possible outcome measure to assess response durability.^1^ NPR after palliative radiation therapy is defined as the proportion of remaining life for which pain is improved and was originally reported by Salazar et al in 1986.^9^ This may be particularly relevant in studies assessing the role of stereotactic treatments. NPR reporting, however, remains rare owing to limitations of data collection and concern about the influence of subsequent treatments upon NPR (as raised by the consensus working party). Where reported, it was approximately 70%,^10^, ^11^ but these studies have not specifically assessed the role of NPR as an outcome.

Although the Dutch Bone Metastasis Study (DBMS) was performed in the late 1990s, the study collected weekly and subsequently monthly response questionnaires on pain and quality of life after radiation therapy.^5^, ^12^ It was a large and well conducted trial, with significant resources invested in minimizing missing data and providing a rich source of longitudinal data. In the current study, we used the DBMS data to assess the role and added value of NPR. We aimed to (1) assess NPR by treatment regimen, diagnostic group, and survival cohorts and (2) carry out sensitivity analyses to assess the consequences of missing data considering the impact this may have upon NPR as an outcome measure.

## Methods and Materials

The data used in this analysis were collected by the DBMS group. Details of the study methods have previously been published^5^, ^12^; however, a brief summary follows. A total of 1157 cancer patients with pain (≥2 on 0-10 numerical rating scale) due to bone metastases from solid tumors were recruited across The Netherlands between March 1996 and September 1998. Patients were randomized to receive a single 8 Gy fraction or 24 Gy in 6 fractions to the painful site. Baseline pain score, analgesia, information about the primary tumor, and demographic characteristics were collected. Individuals were asked to complete weekly follow-up questionnaires for the first 12 weeks, with monthly questionnaires thereafter for a maximum of 24 months (a maximum of 34 questionnaires per patient). Reirradiation data were collected from treating institutes. The database was updated and closed in December 1998. Response rates after treatment have previously been reported.^12^, ^13^, ^14^

For the present analysis, patients were grouped according to their primary tumor into 4 groups: breast cancer, prostate cancer, lung cancer, and all other diagnoses. Baseline characteristics were described for the study population. Survival was defined from the start of radiation therapy. Survival cohorts were identified as individuals surviving <6 weeks, 6 to 12 weeks, 12 to 24 weeks, 24 to 52 weeks, and >52 weeks. Individuals censored at trial closure had unknown survival time. Median survival was calculated using the Kaplan-Meier method.

The patient's pain response at each questionnaire was classified using International Consensus endpoints,^1^ therefore, adjusting for changes in opioid intake (PP, NR, PR, or CR). Each patient's overall best response was defined. Unlike in previous reports, only questionnaire responses after treatment start were considered, rather than those after randomization. No exclusions were made for individuals with missing data to ensure the subsequent sensitivity analyses encompassed all individuals. Each questionnaire was assumed to represent the whole period since treatment or the prior questionnaire. Given the fluctuating nature of pain, it was accepted that patients could move between response states, experiencing initial response, followed by NR, and subsequent return to response. Adjustment for analgesia was made, and this allows for the fluctuating nature of pain an individual may experience while recognizing if this pain improvement was the result of increased analgesia. NPR was then calculated as the sum of time spent with pain response divided by the total time represented by all observed questionnaires (including those beyond reirradiation) and multiplied by 100 to give a percentage (complete questionnaire analysis NPR [cqaNPR]). Calculation of mean cqaNPR was carried out in the following cohorts:

• Initial analyses included only individuals with known survival time who were known to have responded to treatment. This determines observed NPR in responders. This was assessed by survival cohort, diagnosis, and treatment regimen.
• Subsequently, this analysis was reproduced including patients with known survival time, but who were not known to have experienced a response to treatment. This provides overall expected NPR for all patients.
• Those individuals censored at trial closure and known to have responded were then considered separately because their response duration beyond trial closure was not known.

Mean cqaNPR for different treatment regimens was compared using 2-sided Student's *t* test in individuals with known survival time.

Studies investigating interventions in patients near the end of life often have significant amounts of missing data.^15^ This does not imply poor study design or conduct.^16^, ^17^ Where complete case analysis is carried out, however, it must be recognized that the act of completing a questionnaire may not be independent of the patient's health state and therefore may result in biased inference.^15^ Sensitivity analyses were conducted to determine maximum and minimum possible outcomes accounting for missing data. These analyses were carried out by survival cohorts, excluding and including those not known to have responded. This ensures that the sensitivity analyses represent the full range of possibilities. Alternative scenarios considered were as follows:

• cqaNPR assumes that response in unobserved questionnaires was as seen in the observed questionnaires (as detailed earlier). This includes response beyond reirradiation.
• NPRa: All unobserved questionnaires were assumed to represent an NR state (worst case estimate).
• NPRb: Unreturned questionnaires were assumed to represent response (an anticipated overly optimistic scenario).
• NPRlv: A last-value-carried-forward approach was used to replace the missing values. In this analysis, an individual's last known pain state was assumed to persist until the next known state or death. NR is assumed before the first known response after radiation therapy.

Finally, the consequences of excluding all responses beyond reirradiation were assessed (ie, assuming these represent response to reirradiation rather than the original treatment; NPRc). The exclusion of these responses provides an assessment of response durability in the absence of reirradiation.

## Results

**Fig. 1:**
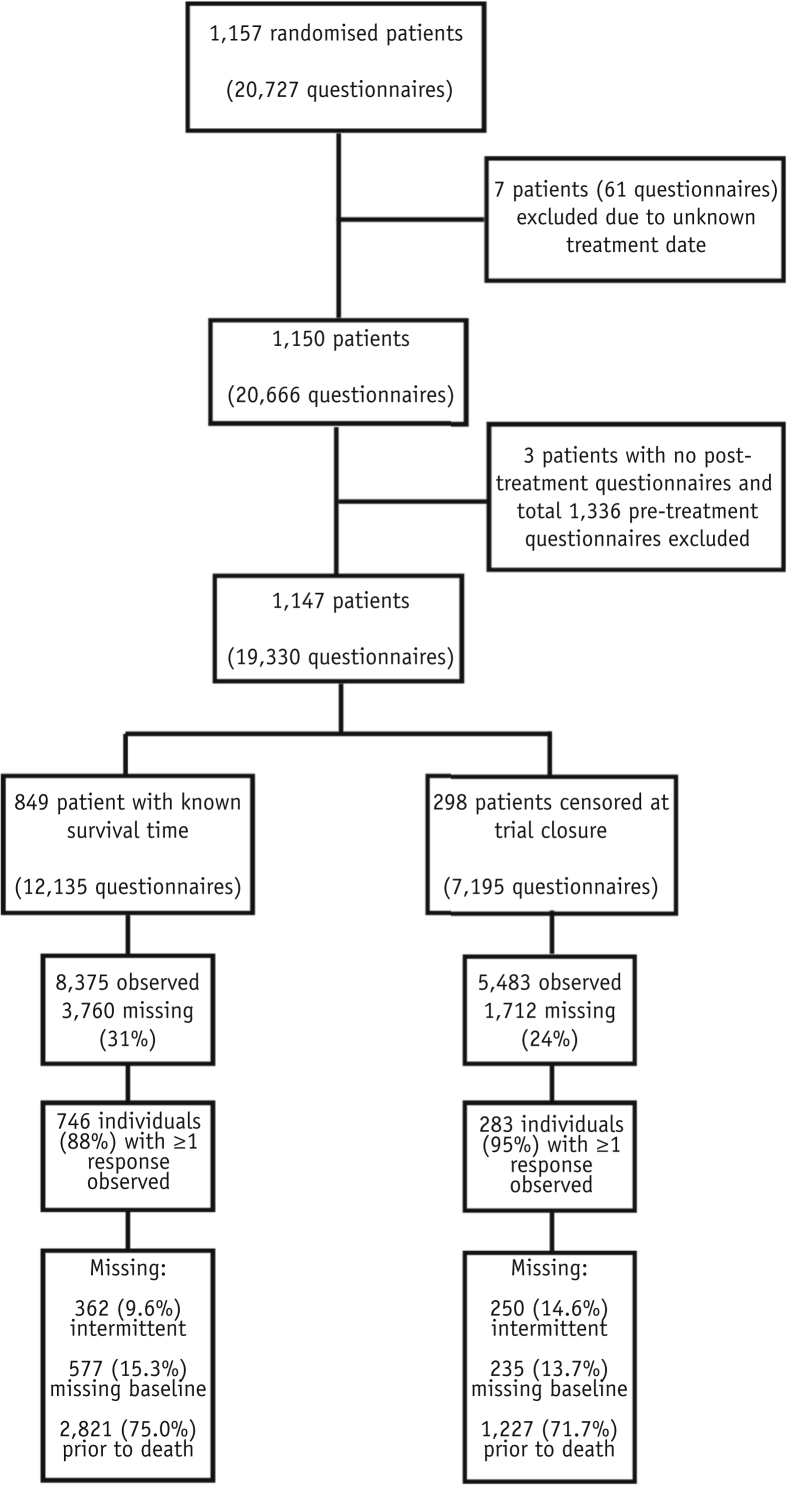
CONSORT diagram illustrating the study population. The 3 patients excluded due to lack of posttreatment questionnaires died before the first follow-up questionnaire was completed.

A total of 1147 patients were included in the study cohort (19,330 questionnaires). Survival time was known in 849 patients (74%) (12,135 questionnaires). The median survival after radiation therapy was 29.6 weeks (26.3-32.9) in the whole population (including those alive at trial closure) and 18.9 weeks (16.9-20.3) in those who died during trial follow-up. Figure 1 demonstrates the identification of the study cohort and details of missing data.

The most common diagnoses were breast (39.2%), lung (24.5%), and prostate (23.2%) cancer. Breast and prostate cancer were more frequent in the survivors beyond study closure. Median baseline pain score was 7 with 26.7% of patients taking strong opiates (World Health Organization pain ladder step 3 or 4).^18^ Baseline characteristics are shown in Table 1.

**Table 1:** Baseline characteristics of the total and the study population

	Total cohort		Final study cohort	
	n	%	n	%
Total	1147	100	849	100
Age (range), y	(32-89)		(32-89)	
<55	239	20.8	169	19.91
55-64	279	24.3	205	24.15
65-74	402	35.1	300	35.34
>74	227	19.8	175	20.61
Sex				
Male	618	53.9	516	60.78
Female	529	46.1	333	39.22
Primary diagnosis				
Breast	449	39.2	261	30.74
Prostate	266	23.2	199	23.44
Lung	281	24.5	257	30.27
Other	151	13.2	132	15.55
Treatment arm				
6 × 4 Gy	571	49.8	430	50.65
1 × 8 Gy	576	50.2	419	49.35
Performance status				
WHO 3-4	170	14.8	155	18.26
WHO 2	436	38.0	359	42.29
WHO 0-1	504	43.9	317	37.34
Unknown	37	3.2	18	2.12
Baseline pain score				
2	16	1.4	7	0.82
3	40	3.5	29	3.42
4	71	6.2	47	5.54
5	131	11.4	91	10.72
6	125	10.9	87	10.25
7	180	15.7	137	16.14
8	330	28.8	257	30.27
9	134	11.7	104	12.25
10	117	10.2	88	10.37
Unknown	3	0.3	2	0.24
Baseline analgesia				
None	145	12.6	86	10.13
Simple	415	36.2	276	32.51
Weak opioids	214	18.7	157	18.49
Strong opioids	306	26.7	273	32.16
Unknown	67	5.8	57	6.71

### Complete questionnaire analysis NPR outcomes

**Fig. 2:**
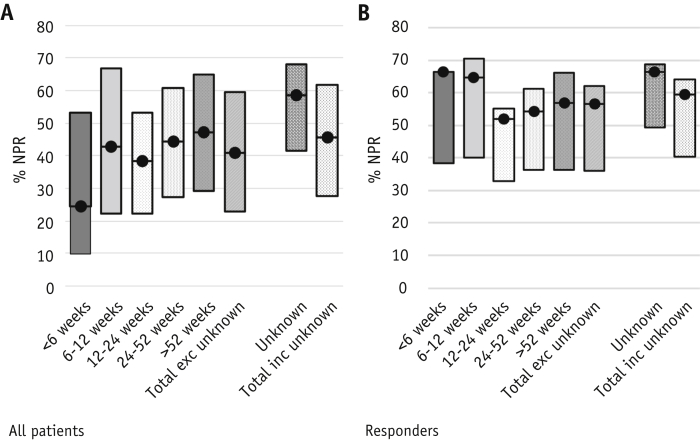
Complete questionnaire analysis net pain relief (NPR) in (a) all patients and (b) responders by survival cohort. Patients censored at trial closure considered separately. Complete questionnaire analysis NPR is shown by the black dot with sensitivity analyses illustrated by surrounding bars (NPRa-NPRb).

**Fig. 3:**
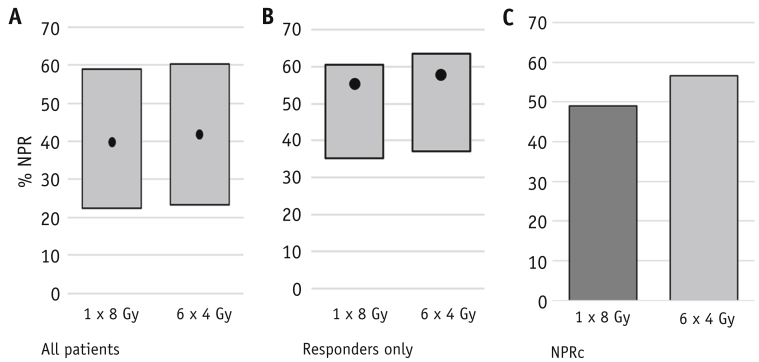
Net pain relief (NPR) by treatment arms; sensitivity analyses shown in the gray bars with complete questionnaire analysis NPR the black dot, sensitivity analyses in gray (NPRa-NPRb), (a) All patients, (b) responders, (c) NPRc–excluding response beyond reirradiation. Individuals censored at trial closure excluded.

Patients responding to radiation therapy with known survival time had a mean cqaNPR of 56.6% (standard error, 1.34) (n = 539), including all responses beyond reirradiation. cqaNPR was higher in those known to have experienced a CR compared with PR (70.2% vs 49.9%, respectively). cqaNPR appeared higher in those with very limited survival, namely 66.4% (<6 weeks of survival [n = 30 patients]) versus 54.4% in long-term survivors (survival >52 weeks [n = 160]), although significantly more data were missing in the short survival cohort, making these results difficult to interpret (Fig. 2a and 2b). When response beyond reirradiation was included, no significant difference was seen in cqaNPR between the 2 treatment regimens (55.4% [single 8 Gy] vs 57.7% [24 Gy in 6 fractions] in responders) (*P* = .191) (Table 2a and Fig. 3). Patients with breast or prostate cancer who responded to treatment experienced higher cqaNPR (59.4%) than those with lung or other cancers (cqaNPR 52.2%) (Table 3).

**Table 2:** Complete questionnaire analysis of net pain relief by radiation therapy regimen received

Arm	% NPR in responders		
(a)	n	Mean (%)	Standard error
1 × 8 Gy	269	55.4	1.95
6 × 4 Gy	270	57.7	1.84
(b)			
1 × 8 Gy	269	49.0	1.88
6 × 4 Gy	270	56.5	2.08

(a) Complete questionnaire analysis of net pain relief values including response beyond re-treatment (*P* = .3863); (b) excluding responses beyond retreatment (*P* = .008). Missing questionnaires accounted for 29.6% and 32.4% in the single 8 Gy and 6 × 4 Gy groups, respectively. Re-treatment occurred after 26.0% of single 8 Gy treatments and 7.9% of 6 × 4 Gy treatments.

**Table 3:** Complete questionnaire analysis of net pain relief by primary diagnosis in patients with known survival time (all patients and those with known pain response considered separately)

Primary diagnosis	All patients			Responders		
	n	Mean (%)	Standard error	n	Mean (%)	Standard error
Breast	236	46.1	*2.42*	183	59.4	*2.33*
Prostate	177	48.6	*2.71*	145	59.3	*2.56*
Lung	215	33.3	*2.41*	134	53.4	*2.63*
Other	118	32.6	*3.18*	77	49.9	*3.52*

In patients alive at study closure who responded to treatment, cqaNPR was 66.3% higher than in those with known survival time (56.6%) (Fig. 2a and 2b). The proportion of breast and prostate cancer patients was higher in those censored at trial closure (85.5% vs 54.2%), potentially contributing to this higher cqaNPR (Table 1).

### Missing data and sensitivity analyses

One hundred and three (12.1%) patients had no observed response assessment after treatment. Median survival of this group was 7.86 weeks (95% confidence interval, 6.57-11.14). In the population who were observed until death, 3760 questionnaires were missing (31.0%). Missing data were more common for patients with limited observed survival (49.4% missing [<6 weeks]; 26.5% missing [>52 weeks]). In individuals who returned at least 1 posttreatment questionnaire, 38.7% of questionnaires were missing in nonresponders whereas 21.6% were missing in responders. Fewer data were missing after single 8 Gy treatment (29.58% vs 32.4% [χ^2^d *P* = .001]).

The outcomes of sensitivity analyses by survival cohort are shown in Figure 2c and 2d. The range of possible outcomes for NPR was 36.1% to 62.1% in responders (cqaNPR 56.6%) and 22.9% to 59.6% (NPRa-NPRb) in all patients (cqaNPR 40.9%). NPRlv revealed outcomes between cqaNPR and the lower limit of sensitivity analyses (52.8% [responders] and 38.1% [all patients]).

Only 115 individuals (13.5%) with known survival time returned enough questionnaires to allow assessment of more than 90% of their remaining life. The median survival of this group was 33.0 weeks; 101 individuals (87.8%) were responders, of whom 48 (41.7%) were complete responders (compared with 17.4% CR in those with <90% time observed). In responders in this cohort the mean cqaNPR was 55.7%.

In patients with known survival time, reirradiation occurred in 26.0% in the single 8 Gy arm and 7.9% in the 24 Gy arm. When all questionnaires beyond reirradiation were assumed to reflect no response, NPRc was greater after 24 Gy than after 8 Gy (56.5% vs 49.0% in responders [*P* = .004]) (Table 2b and Fig. 3c).

## Discussion

Durability of pain control is an important outcome for patients undergoing palliative radiation therapy for painful bone metastases.^19^, ^20^ International consensus endpoints recommend reporting responding proportion; however, this method does not provide information on response durability. Reirradiation rates are used as a proxy for response duration on the assumption that reirradiation reflects PP after an initial pain response. This assumption is questionable when comparing the 2 treatment arms here due to knowledge that reirradiation is carried out at lower pain scores more promptly and in greater numbers irrespective of response status after single 8 Gy treatments compared with 24 Gy in 6 fractions; it is not purely a measure of response durability.^5^ NPR reflects the time spent in response divided by the actual survival time and has potential to provide this information.

In this study, patients who responded to palliative radiation therapy gained improved pain control for 56.6% of their remaining life (cqaNPR). No difference was seen in cqaNPR after single- and multiple-fraction palliative radiation therapy in responders (55.5% vs 57.5%), although a significant difference was seen when response beyond reirradiation was excluded (49.0% vs 56.5% respectively). Although statistically significant, it is unclear if this latter difference is of sufficient scale to be clinically relevant given the recognition that reirradiation is not a simple reflection of pain; therefore, this outcome is likely to be confounded by differential willingness to re-treat between the treatment arms.^5^

### Challenges to NPR as a trial outcome measure

Although appealing in its potential to offer valuable information about response durability, we demonstrate that challenges exist in the use of NPR as an outcome measure.

Despite being a well-conducted study, significant numbers of questionnaire responses are missing, as is anticipated in studies investigating palliative interventions.^15^ Complete questionnaire analysis (cqaNPR) assumes that the proportion of time spent in response in observed questionnaires reflects the proportion in unobserved questionnaires. This assumption is questionable if patients whose clinical condition is below average are less likely to return questionnaires; that more missing data are seen near the end of life reinforces the likely violation of this assumption. As a consequence, cqaNPR is likely to represent an optimistic estimate of true NPR. Those with known survival time and >90% of their remaining life observed (n = 115) provide the greatest certainty in NPR outcomes. In this group, cqaNPR was 55.9%, although the higher proportion of CR in this cohort means this too may be an optimistic estimate. Those who gain a pain response after palliative radiation therapy for bone metastases can expect improved pain control for approximately half of their remaining life, with patients with prostate and breast cancer experiencing better outcomes than those with other cancers.

By reporting both intention to treat and assessable responses, existing studies recognize the impact of missing data (due to death and incomplete data).^2^, ^4^, ^21^ A range of possible outcomes can be derived based on the findings of Chow et al.^2^ Response in assessable individuals (complete case analysis) was 72%, with a possible range of 60% to 76.7%. The range of NPR outcomes demonstrated here (36.1%-62.1%) is greater than that seen in overall response rates. NPR was similar in the treatment groups considered here (8 Gy and 24 Gy); however, if interventions with markedly different outcomes are compared, the potential for systematic bias due to missingness must be acknowledged.

NPR, as measured here, recognizes that patients’ symptoms may fluctuate,^22^ accepting all pain response beyond first recurrence of pain as reflecting this fluctuation. Importantly, where response beyond further treatment (such as reirradiation) was included, the distinction between response due to the initial treatment and response due to subsequent treatment was not made. This is potentially a limitation. It is, however, notable that even where response beyond reirradiation is excluded, the difference between the 2 treatment arms is 7.5% of remaining life in responders. Reirradiation after single 8 Gy treatment was observed in 26% of patients with a known date of death in this study (compared with 7.9% after 24 Gy in 6 fractions). More contemporaneous studies have demonstrated markedly lower reirradiation rates after single 8 Gy treatments (as low as 14%).^23^ Given that the decision to reirradiate is not a simple reflection of pain control, the exclusion of responses post-reirradiation risks introducing the same bias as is present when reirradiation itself is used as an end-point.^5^ Conversely, inclusion of these responses is questionable when assessing response durability. Overall, the proportion of remaining life spent with pain response was the same for patients receiving these 2 regimens. For a proportion of patients undergoing single-fraction treatment, reirradiation may be contributing to this. Notably, 74% of patients in the single-fraction arm did not require reirradiation and were saved a further 5 treatment attendances. Reporting of NPR, both including and excluding response beyond reirradiation, may therefore be appropriate. In addition, future studies should ensure clear protocols to ensure that the reirradiation endpoint is as unbiased as possible.^23^

Patients who responded and were alive at trial closure experienced higher NPR than those observed until death (cqaNPR of 66.3% vs 56.6%). It is not possible to determine whether this difference reflects the nature of the censored group (greater proportion of breast and prostate cancer, longer survival times) or the fact that the observations included were relatively closer to treatment, possibly not including the point of pain recurrence. With well-balanced treatment arms, this may not be a problem, but differing follow-up periods with variable time points for response assessment will limit comparability of NPR between studies. This has implications for the use of NPR in stereotactic radiation therapy studies, as benefit may be anticipated to be greatest in patients with longer survival who are more likely to be censored at trial closure.

A further limitation of the NPR outcome is that a returned questionnaire is assumed to be representative of the entire period since the previous questionnaire. Questionnaires, however, focused upon an individual's worst pain over the preceding week because robust capture of experienced pain can only be achieved over short periods in cancer patients.^12^, ^24^, ^25^ As such, these outcomes rely upon the assumption that symptoms remain stable between questionnaires. Notably, Foro Arnalot et al report NPR of 68%, whereas Salazar et al report NPR of 71%, possibly reflecting the limited longitudinal data collected.^10^, ^11^ Longer periods between questionnaires will increase uncertainty in NPR outcomes.

### Implications for the use of NPR in future bone metastases trials

Unfortunately, missing data during follow-up are inevitable in this patient population and will remain a limitation of NPR as an outcome measure, particularly in subgroups where missing data are more prevalent (eg, those with very limited prognosis). Caution is required in interpreting outcomes in these groups. These uncertainties should not, however, prevent the use of NPR as an outcome measure in future trials, although they have implications for trial conduct and reporting:

• Specific efforts, beyond those already in place, are required to minimize missing data (eg, ensuring complete collection of baseline data, increasing questionnaire completion, and balancing questionnaire burden with adequate follow-up).^15^
• The collection of outcomes using digital platforms has been shown to be feasible and beneficial in advanced cancer and might enable capture of weekly outcomes to reduce uncertainty in the measurement of NPR.^26^
• The use of cohort multiple randomized controlled trial designs could be considered to aid recruitment and gain follow-up information for a cohort receiving standard treatment. The challenges of this in a population with limited prognosis may, however, be significant.^27^, ^28^
• Robust reporting of the extent of missing data and sensitivity analyses will be required to allow comparisons of NPR both within and between trials.^29^
• Although assessing NPR in all patients (both responders and nonresponders) provides an important sensitivity analysis, the increased uncertainty introduced by including nonresponders can be avoided by assessing this outcome only in responders. The question answered by these 2 results differs and given the overall aim of comparing treatment efficacy, it may be necessary to accept (and recognize) this uncertainty.

Despite comparable response rates after fractionated and single-dose treatments for patients with painful bone metastases, the justification offered for fractionated radiation therapy is often response durability, as measured by reirradiation rates.^20^ Yet reirradiation is not simply a function of pain recurrence,^5^ and no measure of durability of pain control has been routinely reported in studies. In addition, if we are to make the case for stereotactic radiation therapy with higher total doses on the basis of durability of pain control, measures that are able to evaluate this outcome are required.

NPR might address these concerns; however, given the inevitability of missing data in palliative care studies, variable frequency of pain assessment, and practicalities of trial duration, uncertainty in the measurement of NPR is significant. Consensus guidelines should consider its incorporation and provide methodological and reporting recommendations to minimize these limitations. If this can be achieved, NPR may provide valuable information within trial information about an outcome of clear importance to patients.^19^, ^30^ Comparison between trials can be improved by consensus but may remain a limitation.

## Conclusions

NPR provides useful additive information next to response percentages when interpreting outcomes of trials treating patients with painful bone metastases.
